# The Michael J. Fox Foundation’s Strategies for Accelerating Translation of LRRK2 into Therapies for Parkinson Disease

**DOI:** 10.3390/cells9081878

**Published:** 2020-08-11

**Authors:** Shalini Padmanabhan, Brian K. Fiske, Marco A.S. Baptista

**Affiliations:** The Michael J. Fox Foundation for Parkinson’s Research, Grand Central Station, P.O. Box 4777, New York, NY 10120, USA

**Keywords:** Parkinson disease, genetic, therapeutic development, Rab, Collaboration, LRRK2, biomarkers, antibodies

## Abstract

Since 2005, The Michael J. Fox Foundation for Parkinson’s Research (MJFF) has invested significant funding and non-funding effort to accelerate research and drug development activity around the Parkinson disease (PD)-associated protein LRRK2. MJFF has spearheaded multiple public/private pre-competitive collaborations that have contributed to our understanding of LRRK2 function; de-risked potential safety questions around the therapeutic use of LRRK2 kinase inhibitors; and generated critical research tools, biosamples, and data for the field. Several LRRK2-targeted therapies are now in human testing due to the hard work of so many in the PD community. In this perspective, we present a holistic description and model of how our Foundation’s support targeted important barriers to LRRK2 research and helped move the field into clinical trials.

## 1. Introduction/Background

Discovered in 2004 [1,2], mutations in the leucine-rich repeat kinase 2 (*LRRK2*) gene represent one of the most common genetic causes of Parkinson disease (PD), explaining an estimated 1–2% of cases overall [3]. In some ethnic groups, in particular those of Ashkenazi Jewish or North African Arab Berber descent, this frequency rises to between 30–40% of cases [4]. Additionally, variants within the *LRRK2* locus are also associated with increased risk for idiopathic PD (iPD) [5,6]. The most common mutation, G2019S, is associated with increased kinase activity of the encoded protein product and cytotoxicity [7]. These findings have fueled strong interest among drug makers to generate LRRK2 kinase inhibitors, now a promising therapeutic strategy in development for PD. 

**Figure 1:**
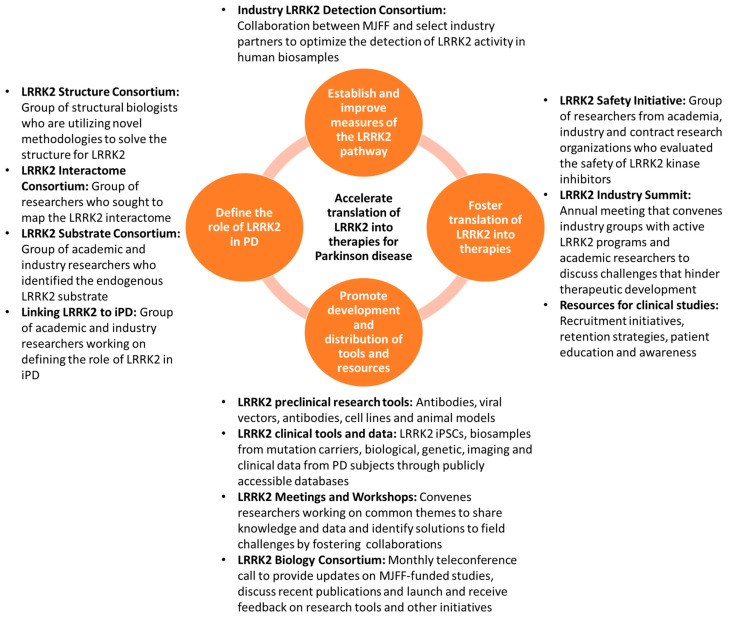
The Michael J. Fox Foundation for Parkinson’s Research (MJFF) applies a “Consortium” approach to tackle key challenges that hinder LRRK2 therapeutic development.

Given its strong genetic link to PD and compelling therapeutic rationale, The Michael J. Fox Foundation for Parkinson’s Research (MJFF) has been a consistent supporter and funder of studies to facilitate translation of LRRK2 research into treatments for people with PD. Our efforts have focused on a multi-faceted strategy with the goal of informing optimal clinical trials of LRRK2-targeted therapies. Through multiple initiatives and programs, MJFF has sought to (1) define the role of LRRK2 in PD; (2) establish and improve measures of the LRRK2 pathway; (3) foster translation of LRRK2 into therapies; and (4) promote development and distribution of tools and resources to facilitate LRRK2 and PD research. To maximize the impact of these strategies and speed delivery of promising treatments to patients, we instilled a philosophy of collaboration and sharing throughout the LRRK2 field by creating and coordinating a broad “Consortium” approach to LRRK2 research and drug development (Figure 1).

## 2. Strategies for Accelerating the Translation of LRRK2 into Therapies for PD

### 2.1. Define the Role of LRRK2 in PD

The nomination of genes, proteins, and pathways potentially involved in PD pathogenesis drives research to characterize these targets and pathways and the cascade of events underlying disease onset, progression and disability. Researchers often need to first clarify the impact of genetic mutations and variations on protein structure and function. This is then expanded by work to define normal and pathological roles of a new target within cells and in more complex biological systems. Ultimately, a mechanistic and pathogenic model linking a protein, its proximal biology, and distal downstream effects on disease becomes clearer, providing validation and support for therapeutic development.

It is through this strategic lens that MJFF established its approach to expanding understanding of the role of LRRK2 in PD. Working closely with a global community of researchers, drug makers, clinicians, and people with and without PD, MJFF has shaped funding strategies to address key questions in the field. We frequently bring key opinion leaders together at scientific meetings and workshops to identify critical gaps and challenges and subsequently find and support expert groups to address these challenges. Below, we describe several key areas where this model has proven particularly successful in furthering our understanding of LRRK2.

First, MJFF has been a leader in supporting work to understand the structure of the LRRK2 protein [8,9,10]. LRRK2 is a large protein containing a kinase domain, a GTPase domain, and additional domains that are crucial for its interaction with other proteins [11,12]. Through the work of an international network of MJFF-supported protein biochemists and crystallographers using multiple approaches, we are peering ever closer at the structural underpinnings of LRRK2. For example, researchers now believe that LRRK2 may shuttle between a monomeric and dimeric state to regulate its activation and function [13,14]. We are also learning more about the complex domain–domain interactions within LRRK2 as well as how associations with other cellular elements, such as microtubules, may act as important regulators of LRRK2 function [15]. Our funding has also supported the first atomic model of LRRK2 [10]. While generating structural information, MJFF-funded teams have developed many useful tools that will further aid in elucidating the full-length structure of LRRK2 and enhance our understanding of its function. In the next few years, we hope to gain even more detailed structural information on the protein along with further information about the intra- and inter-molecular interactions that regulate LRRK2 function. This will pave the way for refinement of current kinase inhibition strategies and development of new strategies for targeting LRRK2.

Second, using a similar collaborative approach, MJFF funded a global team of scientists and drug makers who had access to critical tools and model systems to identify and validate major downstream substrates of LRRK2. This study resulted in the identification of a set of Ras-related in brain (Rab) GTPases as endogenous LRRK2 substrates [16], a finding that laid the groundwork for subsequent initiatives exploring the role of Rab proteins in PD neurodegeneration [17,18,19] and resulted in the generation of critical laboratory tools and reagents that are being distributed across the world to enable further studies of this emerging cellular pathway.

Finally, MJFF support aims to define the upstream modifiers of LRRK2 activation and downstream mechanisms of LRRK2-mediated neurodegeneration using novel methods and model systems. These studies are yielding interesting data on other proteins and pathways that could be targeted to decrease the pathogenic effects of LRRK2 [20,21]. Moreover, MJFF is working with field experts to determine the possible role of LRRK2 in more common, idiopathic forms of PD. As with prior work, we have leveraged a collaborative approach in order to expedite data generation and sharing. Initial evidence points to a possible role for LRRK2 in sporadic PD [5,6,22]. Additional studies will clarify whether LRRK2 therapies may benefit a larger portion of the PD population.

### 2.2. Establish and Improve Measures of the LRRK2 Pathway

Objective biomarkers of LRRK2 protein expression and activity can speed current therapeutic development and incentivize additional industry groups to start new drug programs against this target. Through multiple approaches, MJFF has supported work with the goal of providing a suite of biochemical assays for use in identifying those in the PD population who might benefit from LRRK2 targeted therapies.

Given many early challenges in methods for isolating and assaying the LRRK2 protein, MJFF launched a precompetitive consortium with industry groups to optimize the detection of LRRK2 and its activation in human biosamples. The group convenes frequently to discuss various assay development efforts for detecting LRRK2 levels and activation. In addition, MJFF provided the group standardized clinical samples to enable head-to-head comparison of various high-throughput detection platforms. These efforts identified changes in LRRK2 activation in G2019S subjects by employing a highly sensitive pS935 LRRK2 assay [23] and demonstrated the potential to observe genotype-dependent shifts in LRRK2 inhibitor potency (based on pS935) in human peripheral blood mononuclear cells (PBMCs) that are likely chemotype specific (www.michaeljfox.org/mjff-scientific-publications-clinician-articles). The Consortium is now discussing methods to detect LRRK2 and its activation in the cerebrospinal fluid (CSF) and in immune cells such as monocytes and neutrophils given the high expression of LRRK2 in these cells [24]. These efforts will lead to better assessments of target engagement in the central nervous system (CNS) and periphery and will eventually guide patient selection for LRRK2 trials.

MJFF-supported investigators recently identified bis(monoacylglycerol)phosphate (BMP) as an important indicator of LRRK2 activity [25]. Results are currently being further validated in MJFF’s landmark observational study, the Parkinson’s Progression Marker Initiative (PPMI), to determine if BMP levels serve as a marker for disease progression. Similarly, assays to assess LRRK2 pathway activation such as total LRRK2 and pRab10 protein levels, mitochondrial DNA damage [26], and blood urate [27] have been optimized. Together, these studies point to clear deficits in the LRRK2 pathway in PD subjects when compared to controls. Further validation in larger, well-characterized cohorts such as PPMI will enable one to correlate these analytes to motor and non-motor features of PD and aid in the identification of biomarkers of therapeutic efficacy and patient stratification.

MJFF is also supporting research using clinical samples to determine the biochemical impact of other disease-linked or associated variants in *LRRK2* (R1441G, G2385R, and R1628P). Although these variants alter LRRK2 kinase activity in cellular studies [28], validation in human biosamples would support potential testing of LRRK2-targeted therapies in a larger pool of variant carriers. Moreover, as some variants such as the G2385R and R1628P are more prevalent in the Asian community [29], MJFF generated and shared detailed protocols and videos for isolating blood cell types to better train sites in China, Malaysia, and Singapore [30].

### 2.3. Foster Translation of LRRK2 into Therapies

In the earliest days of research on LRRK2, it was evident that the medicinal chemistry understanding to develop therapies targeting the protein’s kinase activity was far ahead of the biological understanding of its role in cells. This is evidenced by numerous publications and patents describing chemical LRRK2 protein inhibitors [31]. Despite the amount of chemical matter generated, many companies faced critical questions about how to preclinically assess these approaches, whether LRRK2 inhibition will be safe in humans and ultimately how to effectively test LRRK2-targeted drugs in the clinic. A key component of MJFF’s collaborative strategy has been to work closely with leading industry players to access tools and knowledge as well as to collaborate on studies that can best inform how to effectively develop LRRK2-targeted therapies.

While the first leading compounds neared the clinic, a critical question arose as to whether LRRK2 inhibitors might lead to side effects or toxicity that would halt progress. Observations in animal models lacking the LRRK2 gene had already suggested that loss of LRRK2 is associated with morphological changes in cells in the kidney and lungs [32,33,34,35]. Importantly, an MJFF-supported study in collaboration with researchers at Genentech in non-human primates revealed that two of the company’s LRRK2 kinase inhibitors induced a similar lung phenotype [36]. However, whether the effect of these inhibitors was due directly to action on LRRK2 or through another “off-target” action was not clear. If it were a real effect, whether the phenotypes were reversible or associated with any functional impact was not known. The findings led many companies, and the field in general, to question whether LRRK2 inhibition was the right approach to bring to clinical testing.

To address this, MJFF established a pre-competitive LRRK2 Safety Initiative involving three pharmaceutical companies (Pfizer, Merck and Genentech with the latter’s LRRK2 assets eventually being obtained by Denali Therapeutics). Demonstrating a clear willingness to tackle a common challenge together, each company agreed to contribute LRRK2 kinase inhibitors of different structural classes in order to address the question of LRRK2 inhibitor safety. Results of the collaborative study showed that the changes seen in lung were indeed due to an apparent direct action on LRRK2. However, these changes were completely reversible after a two-week washout of drug and the group found no lung functional consequences despite the high drug exposures achieved [37]. In a separate MJFF-supported effort, investigators reported that people who carry heterozygous loss-of-function LRRK2 variants, leading to reduced LRRK2 protein levels, do not have reduced life expectancy, nor show more specific disease-related phenotypes [38]. While additional questions may remain about safety of targeting LRRK2, for example, monitoring immune function is clearly important given the role LRRK2 appears to play in this pathway [39,40,41,42], data from these MJFF-enabled efforts increased confidence for moving LRRK2 inhibitors into human testing.

At the time of this writing, two companies are testing LRRK2 treatments in human trials. Denali Therapeutics was the first company to enter the clinic with a LRRK2 kinase inhibitor in 2017. DNL201, a small molecule LRRK2 inhibitor, completed Phase 1 studies (NCT03710707) and was deemed safe and well-tolerated. Importantly, the company reported no clinically meaningful changes or dose-related trends in the functional pulmonary readouts they tested. Denali Therapeutics is also testing in phase 1b trials another oral LRRK2 inhibitor, DNL151 (NCT04056689). Based on results of these trials, Denali Therapeutics has recently announced that they will be moving forward with DNL151 due to its pharmacokinetic properties and in an exciting new development has partnered with Biogen to develop and commercialize its LRRK2 kinase inhibitor (https://investors.biogen.com/news-releases/news-release-details/biogen-and-denali-collaborate-lrrk2-program-parkinsons-disease).

Along with co-developing a LRRK2 kinase inhibitor with Denali, Biogen Inc is exploring an antisense oligonucleotide approach to decrease brain levels of LRRK2 via administration into the CSF. The company has demonstrated that reduction of LRRK2 levels using this approach ameliorates alpha-synuclein inclusion formation in preclinical models [43]. One advantage of the antisense oligonucleotide approach for targeting LRRK2 is that the inhibition is primarily confined to the CNS, which should minimize possible effects on peripheral organs such as the lung. Recently, the company formally launched a clinical study to evaluate the safety, tolerability, and pharmacokinetics of its antisense product BIIB094 in PD patients (NCT03976349).

Although it is exciting to see these new treatments being tested in humans, it is crucial to keep the LRRK2 therapeutic pipeline robust and diverse. Success rates for drugs in clinical trials for CNS disorders are less than 8% [44]. Therefore, evaluating other ways to manipulate LRRK2 may prove beneficial if first-generation LRRK2 inhibitors prove unsuccessful. For example, targeting other enzymatic and non-enzymatic domains of LRRK2 might result in greater efficacy and fewer side-effects. It may also be worthwhile to move beyond LRRK2 itself and target other components of the LRRK2-related cellular pathway. MJFF is currently funding groups to screen drugs against the specific LRRK2 phosphatase that dephosphorylates T73 Rab10 based on a recent discovery demonstrating that PPM1H phosphatase counteracts LRRK2 signaling [20]. These innovative approaches, although high-risk, may prove to be promising treatment alternatives in the future.

With three LRRK2-targeted therapies in clinical testing and additional therapies likely to move into trials soon, a big challenge will be identifying and recruiting sufficient numbers of G2019S *LRRK2* carriers for Phase II clinical trials. Although G2019S is the most common LRRK2 mutation in some human populations, lack of robust and sensitive PD endpoints requires trials to recruit large numbers of subjects for each Phase II clinical trial. To address this need, various groups, including MJFF, are developing and leveraging strategies to accelerate identification of pools of study volunteers for LRRK2 clinical trials. Through our online survey platform, Fox Insight, MJFF is collaborating with the company 23andMe to genotype more people with PD who may be eligible for LRRK2 trials (www.23andme.com/pd). This work complements the efforts of other organizations who have also launched programs to enhance capacity for finding and educating people with genetic forms of PD (www.parkinson.org/PDGENEration). We are also working to identify people who carry other mutations and variants in LRRK2 in Spain, China, Singapore, and Malaysia. Biosamples from these individuals will be important for further confirming the potential benefits of LRRK2 inhibitors and establishing novel biomarkers. Finally, MJFF is piloting studies within the US for engaging PD patients of Asian descent in genetics research. These efforts are not only addressing recruitment challenges for LRRK2 trials but are also educating and empowering a more diverse and inclusive patient community to participate and contribute to research.

### 2.4. Promote Development and Distribution of Tools and Resources to Facilitate PD Research

In addition to funding, MJFF has long been a leader in providing the community with critical resources such as preclinical tools and models as well as clinical data from cohorts of people with PD and access to human biosamples. MJFF works with contract research organizations and a global network of investigators to generate, distribute and, when needed, further characterize these tools. Through periodic input from the research community via surveys and meetings, we continuously monitor the need for and prioritize those tools that address key gaps in the PD field and speed progress. Through such efforts, the Foundation has made available and continues to generate multiple animal models, antibodies, cell lines, immunoassays, and viral vectors, to study the function of LRRK2 in various cellular and in vivo systems (www.michaeljfox.org/research-tools-catalog).

As part of our PPMI study (www.michaeljfox.org/ppmi-clinical-study), we are following 156 people with PD carrying a LRRK2 mutation and 216 non-manifesting carriers. These volunteers are contributing valuable clinical data and biosamples longitudinally to guide biomarker studies for PD. Through PPMI, the Foundation has made available fibroblasts and induced pluripotent stem cells (iPSCs) from LRRK2 manifesting and non-manifesting carriers that are being used by laboratories around the world for exploring and validating LRRK2′s biological function (www.ppmi-info.org/access-data-specimens/request-pbmcs-cell-lines). With new data emerging on the role of LRRK2 in specialized cells, future efforts are directed at generating isogenic controls for these lines and differentiating these iPSCs into various cell types such as dopamine neurons, astrocytes, and microglia to compare the cell-type specificity of observed LRRK2 effects. Data generated through PPMI and other cohorts are readily available through various platforms for access by researchers that in turn encourages generation of new hypotheses and data standardization and replication (www.michaeljfox.org/data-sets).

MJFF has also strived to provide standardized and high-quality biosamples from human *LRRK2* mutation carriers to facilitate biomarker discovery, optimization, and validation efforts (www.michaeljfox.org/biospecimens). The Foundation works closely with academic laboratories and industry groups to guide sample selection and enforces data return and dissemination of results through MJFF-led calls. These efforts have proved to be extremely beneficial for drug developers as it provides them with the opportunity to leverage our biosample collections for biomarker studies and has benefited the community as they can apply lessons learned from these studies in their laboratories to validate these findings.

Importantly, in addition to being a resource provider, MJFF has encouraged and incentivized progress through effective models of collaboration between key academic and industry stakeholders. MJFF is a strong advocate for open science policies, requiring data and tools generated through our funding to be made available and when possible deposited in publicly accessible repositories. The Foundation frequently convenes stakeholders to evaluate progress and challenges in the field and to revisit and reshape our strategies as needed. We are also aligning our efforts with many other organizations and funders to promote greater transparency in research results through promotion of open-access publication and archiving models. These approaches have been instrumental to success in advancing the LRRK2 field and have provided a model that MJFF now uses across many internal strategic priorities.

## 3. Conclusions

In the last 15 years, the LRRK2 field has overcome challenges in understanding of a complex, disease-associated protein. As a leading strategic funder and facilitator of PD research and drug development, MJFF has used a highly collaborative model to support work around key challenges and barriers to progress. The outcomes of these efforts are evident in the growing number of LRRK2 therapeutic programs moving closer to or currently in clinical trials. MJFF remains dedicated to fueling and fostering the drug discovery pipeline for PD with the goal of bringing new therapies into the hands of patients.
